# GPIHBP1 and ANGPTL4 Utilize Protein Disorder to Orchestrate Order in Plasma Triglyceride Metabolism and Regulate Compartmentalization of LPL Activity

**DOI:** 10.3389/fcell.2021.702508

**Published:** 2021-07-15

**Authors:** Kristian Kølby Kristensen, Katrine Zinck Leth-Espensen, Anni Kumari, Anne Louise Grønnemose, Anne-Marie Lund-Winther, Stephen G. Young, Michael Ploug

**Affiliations:** ^1^Finsen Laboratory, Rigshospitalet, Copenhagen, Denmark; ^2^Biotech Research and Innovation Centre, University of Copenhagen, Copenhagen, Denmark; ^3^Departments of Medicine, David Geffen School of Medicine, University of California, Los Angeles, Los Angeles, CA, United States; ^4^Department of Human Genetics, David Geffen School of Medicine, University of California, Los Angeles, Los Angeles, CA, United States

**Keywords:** GPIHBP1, lipoprotein lipase, intravascular lipolysis, intrinsic disorder, ANGPTL4, LU domain

## Abstract

Intravascular processing of triglyceride-rich lipoproteins (TRLs) is crucial for delivery of dietary lipids fueling energy metabolism in heart and skeletal muscle and for storage in white adipose tissue. During the last decade, mechanisms underlying focal lipolytic processing of TRLs along the luminal surface of capillaries have been clarified by fresh insights into the functions of lipoprotein lipase (LPL); LPL’s dedicated transporter protein, glycosylphosphatidylinositol-anchored high density lipoprotein–binding protein 1 (GPIHBP1); and its endogenous inhibitors, angiopoietin-like (ANGPTL) proteins 3, 4, and 8. Key discoveries in LPL biology include solving the crystal structure of LPL, showing LPL is catalytically active as a monomer rather than as a homodimer, and that the borderline stability of LPL’s hydrolase domain is crucial for the regulation of LPL activity. Another key discovery was understanding how ANGPTL4 regulates LPL activity. The binding of ANGPTL4 to LPL sequences adjacent to the catalytic cavity triggers cooperative and sequential unfolding of LPL’s hydrolase domain resulting in irreversible collapse of the catalytic cavity and loss of LPL activity. Recent studies have highlighted the importance of the ANGPTL3–ANGPTL8 complex for endocrine regulation of LPL activity in oxidative organs (e.g., heart, skeletal muscle, brown adipose tissue), but the molecular mechanisms have not been fully defined. New insights have also been gained into LPL–GPIHBP1 interactions and how GPIHBP1 moves LPL to its site of action in the capillary lumen. GPIHBP1 is an atypical member of the LU (Ly6/uPAR) domain protein superfamily, containing an intrinsically disordered and highly acidic N-terminal extension and a disulfide bond–rich three-fingered LU domain. Both the disordered acidic domain and the folded LU domain are crucial for the stability and transport of LPL, and for modulating its susceptibility to ANGPTL4-mediated unfolding. This review focuses on recent advances in the biology and biochemistry of crucial proteins for intravascular lipolysis.

## Introduction

Dietary lipids are absorbed by the intestinal epithelium and packaged into triglyceride-rich lipoprotein (TRLs) called chylomicrons (Dash et al., 2015). Another type of TRLs called very low-density lipoproteins (VLDL) are produced in the liver (Heeren and Scheja, 2021). Newly secreted TRLs enter into the bloodstream and ultimately marginate along the luminal surfaces of capillaries, where triglycerides are hydrolyzed by lipoprotein lipase (LPL). This process releases free fatty acids and monoacylglycerol for use as fuel in heart, skeletal muscle and brown adipose tissue or for storage in white adipose tissue (WAT). Genetic studies revealed that homozygous or biallelic loss-of-function variants in *LPL* or its partners (*GPIHBP1*, *APOC2, LMF1, APOA5*) severely impair the efficiency of triglyceride hydrolysis, causing lifelong severe hypertriglyceridemia—the familial chylomicronemia syndrome (Dron and Hegele, 2020). Chylomicronemia markedly increases risk for debilitating and life-threatening bouts of acute pancreatitis (Goldberg and Chait, 2020; Hansen et al., 2020). Heterozygous loss of LPL causes milder increases in plasma triglyceride levels, which are associated with increased risk for atherosclerotic cardiovascular disease (ASCVD) (Khera et al., 2017). In contrast to the adverse effects of impaired TLR processing, increased efficiency of TRL processing is associated with lower plasma triglyceride levels and reduced risk of ASCVD (Crosby et al., 2014; Jorgensen et al., 2014; Dewey et al., 2017; Graham et al., 2017). For example, genetic variants of angiopoietin-like (ANGPTL) proteins 3 and 4 with a reduced capacity for LPL inhibition are associated with decreased risk of ASCVD (Dewey et al., 2016; Helgadottir et al., 2016; van Leeuwen et al., 2016; Stitziel et al., 2017; Klarin et al., 2018; Hahn et al., 2020; Wang Q. et al., 2020; Wang Z. et al., 2020). Similarly, loss-of-function mutations in *APOC3* accelerate TRL processing and reduce risk of ASCVD (Pollin et al., 2008; Jorgensen et al., 2014).

**FIGURE 1:**
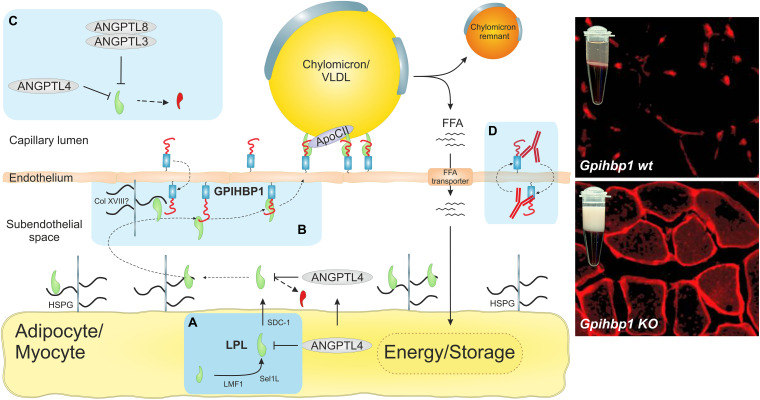
The intravascular unit supports focal triglyceride hydrolysis in capillaries. The light blue boxes highlight recent discoveries concerning LPL-mediated triglyceride hydrolysis in capillaries. **(A)** Recent data suggested that LPL is synthesized and secreted as a monomer rather that the widely assumed head-to-tail homodimer (Beigneux et al., 2019; Kristensen et al., 2020a). Because LPL is inherently unstable (Leth-Espensen et al., 2021), it must be chaperoned in all compartments to maintain its native fold. During biosynthesis by parenchymal cells, LPL is chaperoned in the ER by lipase maturation factor 1 (LMF1) and Sel-1 suppressor of Lin-12-like 1 (Sel1L). In the secretory pathway after transit from the *trans*-Golgi network, LPL is chaperoned by heparan sulfate–modified syndecan-1 (SDC1) (Sundberg et al., 2019). In the subendothelial space, LPL is stabilized by heparan sulfate proteoglycans (HSPGs) in the extracellular matrix and in the glycocalyx of parenchymal cells. **(B)** The seminal discovery that GPIHBP1 shuttles LPL from the abluminal endothelial surface to its site of action in the capillary lumen (Beigneux et al., 2007; Davies et al., 2010; Goulbourne et al., 2014) solved a longstanding enigma (Young et al., 2019). The immunofluorescence micrographs show that LPL (*red*) is localized within capillaries in wild-type mice, but remains mislocalized in the subendothelial space of GPIHBP1-deficient mice, which develop severe chylomicronemia with creamy TRL-rich plasma. GPIHBP1 is organized as a functional dipole with an N-terminal intrinsically disordered acidic domain, which is tethered to a folded disulfide-rich LU domain that harbors a C-terminal glycolipid anchor (Fong et al., 2016; Mysling et al., 2016a). This unique architecture renders GPIHBP1 an efficient chaperone for LPL, stabilizing its native and active conformation (Mysling et al., 2016a). The acidic domain increases the association rate of GPIHBP1 and LPL by 2500-fold, which enables the transition of LPL from an HSPG-bound state to a GPIHBP1-bound state and its subsequent transcytosis to the capillary lumen (Kristensen et al., 2018). **(C)** Inhibition of LPL activity by ANGPTL-3, -4, and -8 was widely assumed to work by converting stable LPL dimers to unstable monomers. HDX-MS studies showed that ANGPTL4 catalyzes the irreversible unfolding of LPL’s α/β-hydrolase domain by directly targeting LPL monomers (Mysling et al., 2016b; Kristensen et al., 2020a, b; Leth-Espensen et al., 2021). Importantly, GPIHBP1 binding counteracts this inhibition of LPL. A oligomeric complex of ANGPTL3 and ANGPTL8 helps regulate LPL activity in oxidative tissues (Chi et al., 2017; Gusarova et al., 2017; Haller et al., 2017; Kovrov et al., 2019; Chen et al., 2020; Oldoni et al., 2020). **(D)** The discovery of inhibitory GPIHBP1 autoantibodies revealed a new etiology of acquired hypertriglyceridemia in some patients without any documented mutations in *LPL*, *GPIHBP1*, *APOC2*, *APOA5*, or *LMF1* (Beigneux et al., 2017; Lutz et al., 2020; Miyashita et al., 2020). The immunofluorescence micrographs were modified and reproduced with permission (Davies et al., 2010).

In the past decade, the prevailing model for LPL-mediated TRL processing has been transformed by new discoveries on the function and dynamics of proteins that participate in LPL transport (GPIHBP1) and help control LPL activity (ANGPTLs). In this review, we focus on the dynamic interplay between LPL, GPIHBP1, and ANGPTLs 3, 4, and 8. We will emphasize the importance of transient interactions between these proteins and discuss the relevance of protein disorder and marginal protein stability to the regulation of LPL and to the compartmentalization of its activity in the intravascular unit. We define the intravascular unit as (i) the capillary endothelial cell, (ii) the subendothelial spaces containing extracellular matrix molecules, and (iii) the parenchymal cells (myocytes and adipocytes) adjacent to capillaries (Figure 1).

## Lipoprotein Lipase

Discovered in 1943 as a heparin-releasable clearing factor (Hahn, 1943), lipoprotein lipase (LPL) was isolated, characterized as a triglyceride hydrolase, and named LPL in 1955 (Korn, 1955). A few years later, LPL deficiency was shown to cause chylomicronemia—the first example of an inborn error in plasma lipid metabolism (Havel and Gordon, 1960). Even though LPL was recognized as the rate-limiting enzyme controlling intravascular triglyceride hydrolysis and relevant to human disease, the protein structure of LPL remained elusive for the next seven decades. Its crystal structure was first solved in 2019 (Birrane et al., 2019) and was confirmed later that year (Arora et al., 2019).

The appearance of LPL in evolution predates teleosts; it is found in all vertebrates and is highly conserved in mammals, with 58–99% sequence identity at the amino acid level (Holmes et al., 2011). LPL is expressed in many tissues, but the highest levels of expression are in parenchymal cells of tissues with robust lipid metabolism (e.g., heart, skeletal muscle, brown adipose tissue) or tissues with a key crucial role in energy storage (e.g., WAT). LPL is also expressed in macrophages, secretory cells of the mammary gland, and hepatocytes of suckling rodents (Kersten, 2014).

### Structure and Stability of LPL

Lipoprotein lipase is secreted as a 55-kDa glycoprotein and belongs to the triglyceride lipase gene subfamily—along with hepatic triglyceride lipase and endothelial lipase. Despite its relatively broad enzyme specificity, LPL primarily hydrolyzes the *sn*-1/*sn*-3 ester bonds of triglycerides in the neutral lipid core of chylomicrons and VLDL, thereby releasing two unesterified fatty acids and a *sn-2* monoacylglycerol (Scow and Olivecrona, 1977). Before the atomic structure of LPL was defined, assumptions about its three-dimensional structure and function were guided by homology models based on the crystal structures of pancreatic lipase (van Tilbeurgh et al., 1994; Kobayashi et al., 2002; Hayne et al., 2018). Homology considerations and biochemical studies indicated that LPL contains two domains: (i) an N-terminal α/β-hydrolase fold harboring the catalytic triad and a lid segment that controls substrate accessibility to the active site and (ii) a C-terminal domain with a β-barrel fold harboring a surface-exposed tryptophan-rich loop that is important for lipid and lipoprotein binding.

#### Crystal Structure of LPL

**FIGURE 2:**
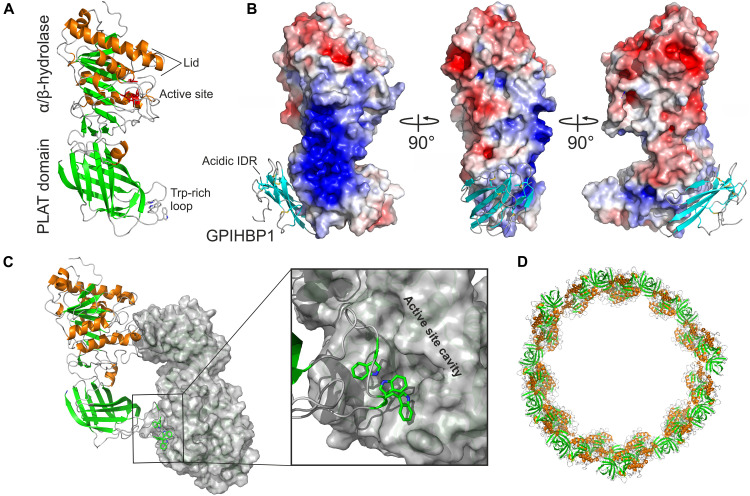
Structure and oligomeric states of LPL. **(A)** The crystal structure of human LPL. β-strands are *green*, α-helices are *orange*, and tryptophan residues in the Trp-rich loop are shown as sticks as defined by *in silico* modeling (Birrane et al., 2019). **(B)** Heparin binding motifs in the LPL sequence fuse in the three-dimensional space to form a large basic patch covering 2400 Å^2^ as illustrated by the electrostatic surface representation (*blue*). The LU domain of GPIHBP1 is bound to LPL’s PLAT domain; β-strands are *cyan*. GPIHBP1’s acidic and intrinsic disordered region (IDR) was not defined by the electron densities, but the position of the N-terminus from the LU domain suggests that the acidic domain projects toward the basic patch of LPL. **(C)** LPL crystalizes as a head-to-tail homodimer. One protomer is shown in a cartoon and the other as a gray surface representation. One of the two reciprocal dimer interfaces is shown in a close-up with the Trp-rich loop of one protomer occluding the active site of the other protomer. **(D)** In the presence of a high salt concentration or heparin, LPL may enter a higher oligomer state and form helical fibers (Gunn et al., 2020). The building blocks of these elongated fibers are the head-to-tail homodimers shown in **(C)**. Illustrations were generated with the PyMol Molecular Graphics System (Schrödinger, LLC) using PDB coordinates 6E7K and 6U7M.

Purified preparations of LPL are notoriously unstable, and excipients such as heparin, high concentrations of sodium chloride, glycerol, sodium deoxycholate, or sodium laurate are required to preserve its activity (Fielding, 1968; Osborne et al., 1985; Lookene et al., 2004; Cheng et al., 2021). This inherent protein instability, along with a propensity for protein aggregation, hampered efforts to define LPL’s three-dimensional structure by X-ray crystallography. That situation changed with the discovery that LPL’s endothelial cell binding partner, GPIHBP1, stabilizes LPL by preventing the α/β-hydrolase domain from unfolding (Beigneux et al., 2007; Mysling et al., 2016a). In short order, two groups reported virtually identical X-ray structures for a LPL•GPIHBP1 complex at 2.8 Å resolution (Arora et al., 2019; Birrane et al., 2019). In line with the prevailing view that LPL is a homodimer, LPL was crystalized as a head-to-tail homodimer (Figure 2C). The N-terminal α/β-hydrolase domain contains six α-helices and 10 β-strands; the C-terminal flattened β-barrel domain adopts a polycystin-1, lipoxygenase, and alpha toxin (PLAT) fold containing 12 β-strands (Figure 2A). The interfaces of the two partner LPL protomers within the head-to-tail LPL homodimer are small (∼600 Å^2^). Of note, the tryptophan-rich loop of one LPL protomer occludes the catalytic cleft of the partner protomer, raising doubts about whether the homodimer configuration in the crystal structure is enzymatically active (Figure 2C). Very likely, the homodimer conformation results from the high protein concentrations required for crystallization and the need to limit solvent exposure to hydrophobic regions of the enzyme.

In the crystal structure (Birrane et al., 2019), LPL’s α/β-hydrolase domain (residues 1–313)^1^ contains a canonical catalytic triad (Ser^132^, Asp^156^, His^241^) with an oxyanion hole (Trp^55^ and Leu^133^) and a lid region (residues 217–238) constrained by a Cys^216^–Cys^239^ disulfide bond. LPL was in an open lid configuration, presumably because the catalytic cleft was occluded by the Trp-rich loop of the PLAT domain (residues 387–394) (Figure 2C). Hydrogen–deuterium exchange mass spectrometry (HDX-MS) indicated that LPL’s lid is highly dynamic—even in LPL monomers (Kristensen et al., 2020a). The crystal structure also revealed that the α/β-hydrolase domain contains a single calcium atom (coordinated by Ala^167^, Ser^172^, Asp^174^, and Asp^175^) required for correct folding *in vivo* and for restoring LPL activity during refolding *in vitro* (Zhang et al., 2005). Interestingly, a missense mutation in one of LPL’s calcium-coordinating amino acids (Asp^174^) had been uncovered in a kindred with chylomicronemia (Abifadel et al., 2004). This missense mutation, p.Asp^174^Val, abolished LPL secretion from cells (Birrane et al., 2019). The crystal structure also revealed that LPL’s heparin-binding motifs are not dispersed within the structure but instead form a large surface exposed basic patch (2,400 Å^2^) spanning the N- and C-terminal domains and extending across the interdomain interface (Figure 2B). This strong cationic region on the surface of LPL plays an essential role in LPL sequestering on heparan sulfate proteoglycans (HSPGs), transport, stability, and regulation in the different compartments within the intravascular unit (Figure 1).

#### Oligomeric States of LPL

Years ago, sedimentation equilibrium analyses of purified preparations of bovine LPL strongly suggested that LPL was a homodimer (Iverius and Ostlund-Lindqvist, 1976; Osborne et al., 1985). Subsequent findings from radiation inactivation experiments (Olivecrona et al., 1985) and immunochemical studies (Zambon et al., 1996) were interpreted as being consistent with homodimers, and the notion that LPL was active as a head-to-tail homodimer gained nearly universal acceptance. Furthermore, the rapid loss of catalytic activity in purified preparations of LPL was widely assumed to reflect dissociation of stable LPL homodimers into unstable and inactive monomers (Lookene et al., 2004; Sukonina et al., 2006). That paradigm persisted for decades and remained largely unchallenged mainly because of difficulties in isolating LPL monomers in concentrations suitable for structural and functional studies.

Recently, Beigneux et al. (2019) revisited the LPL homodimer paradigm using the same biochemical and immunochemical approaches used earlier, but rather than examining purified LPL preparations they analyzed freshly secreted LPL in the medium of cultured CHO cells. Under their cell culture conditions (i.e., low concentrations of LPL, normal ion strength, and absence of heparin), LPL was monomeric and highly active. Subsequent HDX-MS studies strongly supported the idea that LPL is a monomer and showed that LPL is stable and active when the propensity for homodimer formation was abolished by an Fab fragment from the LPL-specific monoclonal antibody 5D2 (Kristensen et al., 2020a). Antibody 5D2 binds the Trp-rich loop in LPL’s C-terminal PLAT domain (Luz et al., 2020), preventing the formation of the head-to-tail LPL homodimers observed in the crystal structure (Arora et al., 2019; Birrane et al., 2019). Thus, all of the LPL was trapped in the monomer conformation, which proved to be both stable and catalytically active, as judged by HDX-MS studies and enzymatic activity assays (using a soluble esterase substrate) (Kristensen et al., 2020a). Of note, LPL homodimers and monomers interconvert *in vitro* as judged by the rapid exchange of LPL protomers under conditions that favor interactions between LPL molecules (Lookene et al., 2004). As shown by cryo-electron microscopy (Gunn et al., 2020), LPL adopts a oligomeric conformation containing helical fibrils composed of head-to-tail LPL homodimers at high protein concentration and low temperature in the presence of high salt and heparin (Figure 2D). Once again, it seems likely that this oligomeric conformation is driven by the need to shield functionally important hydrophobic regions within LPL—the lid and substrate binding pocket in the hydrolase domain and the Trp-rich lipid-binding motif in the C-terminal PLAT domain—from solvent exposure. The resultant propensity of LPL for reversible dimerization *in vitro* may likely have added to the ambiguity regarding the biologically relevant state of LPL.

#### Borderline Protein Stability of LPL

Purified LPL spontaneously loses its catalytic activity in a time-, concentration-, and temperature-dependent manner. Historically, this instability has generally been ascribed to dissociation of active LPL homodimers into inactive monomers (Osborne et al., 1985; Lookene et al., 2004). Recent biophysical studies have provided fresh insights into the molecular mechanisms for the inherent instability of LPL (Mysling et al., 2016a; Leth-Espensen et al., 2021). First, studies by HDX-MS of purified LPL showed that large parts of LPL’s α/β-hydrolase domain (which harbors the catalytic triad) undergo spontaneous and irreversible unfolding, as shown by the emergence of bimodal isotope envelopes after pulse-labeling in deuterium oxide (Mysling et al., 2016a). This unfolding was mirrored by loss of lipase activity. Second, differential scanning fluorimetry revealed that LPL’s N-terminal α/β-hydrolase domain is extremely unstable, with an apparent melting temperature (*T*_m_) of 34.8°C—below normal body temperature (Leth-Espensen et al., 2021). In contrast, LPL’s C-terminal PLAT domain is highly stable with a *T*_m_ of 64.7°C. These findings imply that free LPL is quite unstable at body temperature and subject to progressive inactivation from unfolding of its catalytic domain. Given the intrinsic instability of purified LPL preparations, what explains the extraordinary efficacy of LPL in hydrolyzing triglycerides *in vivo*? The answer is that LPL is likely chaperoned by stabilizing binding partners in each compartment of the intravascular unit.

### Chaperoning of Nascent LPL During Synthesis and Secretion

Because of its intrinsic instability and aggregation prone nature, LPL needs to be stabilized during its biosynthesis, transport, and secretion by a dedicated set of chaperones and transport proteins (Ellgaard and Helenius, 2003). Furthermore, unfolded and aggregated LPL needs to be recognized and removed by ER-associated degradation in the proteasomes or autophagosomes (An et al., 2017). Interestingly, in pulse-chase experiments ∼80% of newly synthesized LPL in adipocytes is not secreted and instead is routed for intracellular degradation (Vannier and Ailhaud, 1989).

Peterfy et al. (2007) reported that lipase maturation factor 1 (LMF1) is required for LPL secretion, and theorized that LMF1 helps assemble inactive LPL monomers into secretion-competent catalytically active homodimers (Koerner et al., 2019). Since LPL is active as a monomer (Beigneux et al., 2019) and LMF1 associates with oxidoreductases and helps to maintain ER redox potential (Roberts et al., 2018), it seems possible that LMF1 helps to ensure proper disulfide formation in LPL monomers. From a practical point of view, increased *LMF1* expression has been reported to increase the expression of recombinant human LPL in CHO cells (Birrane et al., 2019). Another ER protein affecting LPL maturation and secretion is Sel1L (Sel-1 suppressor of Lin-12-like 1), an adapter protein for an E3 ligase in the ER-associated degradation pathway. In Sel1L deficiency, LPL remains trapped in the ER in the form of unfolded aggregates, which are removed by autophagy (Sha et al., 2014). Perhaps LMF1 helps folding LPL monomers while Sel1L ensures that only properly folded LPL exits the ER and is secreted from cells (Wu et al., 2021). Because LPL contains two N-linked glycans (on Asn^43^ and Asn^359^), the calnexin and calreticulin cycle probably also plays a role in LPL quality control (Ellgaard and Helenius, 2003).

After exiting the ER, properly folded LPL is further processed in the Golgi before escaping via the *trans*-Golgi network into the secretory pathway. In elegant studies, Sundberg et al. (2019) demonstrated that LPL interacts with the integral membrane protein syndecan-1 (SDC-1) in the Golgi and then enters a sphingomyelin-enriched vesicular sorting pathway for its secretion. Intriguingly, LPL needs to engage SDC-1, which acts as the obligate sorting receptor for LPL and drives LPL secretion via the sphingomyelin-enriched pathway. SDC-1 is a HSPG, and binding of its negatively charged heparan sulfates to LPL’s positively charged heparin-binding motifs is essential for LPL secretion. Because even small sized heparin fragments improve LPL stability *in vitro* and increases the *T*_m_ from 34.8 to 42.2°C (Leth-Espensen et al., 2021), we suspect that SDC-1 has a dual role in LPL secretion. First, it serves as a sorting receptor in the Golgi. Second, the binding of its glycosaminoglycans to LPL’s large basic patch likely stabilizes LPL and protects its α/β-hydrolase domain from unfolding. Sundberg et al. (2019) also found that LPL’s C-terminal Trp-rich loop is required for secretion. The hydrophobic Trp-rich loop appears to interact with the membrane of sphingomyelin-rich secretory vesicles. We hypothesize, that the anchoring of LPL’s C-terminal domain to membrane lipids and the N-terminal anchoring provided by SDC-1 stabilizes LPL by limiting flexibility between its N-terminal α/β-hydrolase domain and its C-terminal PLAT domain. This stabilization of LPL in the secretory pathway is reminiscent of the stabilization provided by GPIHBP1 on capillary endothelial cells, where GPIHBP1’s acidic domain interacts electrostatically with LPL’s large basic patch and its LU domain engages LPL’s PLAT domain by hydrophobic contacts. This two-point tethering of LPL by GPIHBP1 confers extremely high thermal stability to LPL’s α/β-hydrolase domain (raising the *T*_m_ to 57.6°C). The functional relevance of dual tethering by SDC-1 and sphingolipid-rich secretory vesicles was supported by experiments with an LPL mutant harboring a dysfunctional Trp-rich loop. Secretion of that LPL mutant was inefficient but was restored when the mutant was co-expressed with GPIHBP1 (Sundberg et al., 2019). Of note, the Trp-rich loop is not important for GPIHBP1 binding.

Another possibility is that the Trp-rich loop of the PLAT domain is required for the assembly of head-to-tail LPL homodimers that are the minimal building blocks in the helical assembly of LPL oligomers. It is possible that these helical LPL oligomers associate with SDC-1 in secretory vesicles (Gunn et al., 2020). Further studies are required to define the conformation of LPL within the secretory pathway. It would be interesting to determine, by cryo-electron microscopy, whether introducing N-linked glycans into the interface between partner LPL *homodimers* would prevent the formation of helical oligomers and, if so, whether that modification would limit LPL’s entry into the sphingomyelin-enriched secretory pathway.

Intracellular trafficking of LPL beyond the *trans*-Golgi network likely includes mechanisms for routing misfolded LPL to an endolysosomal pathway for lysosomal degradation. Apart from a reported interaction between LPL’s PLAT domain and the sortilin-related receptor (Klinger et al., 2011), the mechanisms responsible for channeling misfolded LPL for disposal are largely unknown. One possible molecular cue that could trigger LPL disposal is unfolding of LPL by ANGPTL4 in the *trans*-Golgi network (Dijk et al., 2016, 2018).

### Disease-Relevant Human LPL Variants

Genetic studies have uncovered more than 100 *LPL* variants, the majority of which are loss-of-function variants in patients with hypertriglyceridemia (Rodrigues et al., 2016). The prevalence of LPL deficiency increases from 1 to 2 per million in the general population to 9 per 1000 in patients with severe hypertriglyceridemia (>20 mmol/L). Many deleterious variants affect functionally important regions of LPL, for example the catalytic triad (e.g., p.Asp^156^Asn, p.Asp^156^His, p.Asp^156^Gly), a Ca^2+^-coordinating amino acid (p.Asp^175^Val), the lid region (e.g., p.Cys^216^Ser, p.Ile^225^Thr), and LPL’s binding interface with GPIHBP1 (e.g., p.Met^337^Arg, p.Cys^418^Tyr). The crystal structure of LPL has been useful in understanding how LPL mutations affect function (Birrane et al., 2019). As noted earlier, we speculate that limiting flexibility between LPL’s α/β-hydrolase and PLAT domains with SDC-1 or GPIHBP1 (*via* a dual interaction with LPL’s basic patch and its PLAT domain) helps stabilize LPL and preserve its structure and activity. Several genetic variants associated with hypertriglyceridemia are located in the interface between the N- and C-terminal domains of LPL (e.g., p.Ser^259^Arg, p.Gly^409^Arg, p.Glu^410^Val).

A common *LPL* polymorphic variant (p.Ser^447^X, with an allele frequency of 12–16%) results in a truncated LPL protein lacking the last two residues (Ser-Gly). Interestingly, this variant has a gain-of-function phenotype characterized by reduced plasma triglyceride levels, increased pre- and post-heparin LPL levels, and reduced risk of cardiovascular disease (Rip et al., 2006). Because of these beneficial properties, the p.Ser^447^X variant has attracted considerable attention. Adenovirus-mediated expression of the p.Ser^447^X variant prevents early perinatal mortality in LPL knockout mice (Ross et al., 2005) and AAV-mediated expression of this variant in humans with LPL deficiency mitigates disease phenotypes (Scott, 2015). The mechanism of the beneficial effects of the p.Ser^447^X variant remains speculative (Rip et al., 2006). One possibility is that the p.Ser^447^X gene variant is in linkage disequilibrium with single-nucleotide polymorphisms that disrupt seed sites for microRNAs downregulating LPL mRNA translation (Richardson et al., 2013; Caussy et al., 2016). The resultant increase in overall LPL expression is likely to add to the gain-of-function phenotype of p.Ser^447^X. Another possibility, is that the p.Ser^447^X variant interacts more efficiently with GPIHBP1’s acidic domain and thereby increases LPL stability. Of note, the p.Ser^447^X variant terminates with Lys-Lys, which is predicted to be positioned close to LPL’s basic patch. We speculate, that the truncation of the p.Ser^447^X variant could remove a small kinetic barrier that slows the interaction with wild-type LPL and that the deletion of Ser-Gly from the C-terminus allows a more extensive electrostatic interaction with GPIHBP1’s acidic domain. Western blotting showed no gross differences in the binding of wild-type LPL and p.Ser^447^X LPL to GPIHBP1 (Turlo et al., 2014), but western blotting would almost certainly be insensitive to subtle kinetic differences in the LPL–GPIHBP1 electrostatic interactions. Surface plasmon resonance would likely be required to determine whether or not p.Ser^447^X LPL associates more effectively with GPIHBP1’s acidic domain.

## Glycosylphosphatidylinositol-Anchored High Density Lipoprotein–Binding Protein 1

Six decades after the discovery of LPL, GPIHBP1 was identified by expression cloning as a GPI-anchored protein that enables transfected cells to bind high density lipoproteins (Ioka et al., 2003). The prime function of GPIHBP1 as the obligate endothelial binding partner for LPL was first reported in 2007 (Beigneux et al., 2007). In several seminal papers, the same group revealed an essential role for GPIHBP1 in intravascular lipid metabolism (Davies et al., 2010, 2012; Goulbourne et al., 2014; Beigneux et al., 2017). GPIHBP1 expression is strictly confined to the capillary endothelium in peripheral tissues and is absent from venules, arterioles, and larger blood vessels (Davies et al., 2010; Meng et al., 2020). GPIHBP1 and LPL expression levels are matched pairwise in most tissues, except the lungs, where GPIHBP1 expression is high and LPL expression is negligible (Olafsen et al., 2010). The role of GPIHBP1 in the lung remains enigmatic, since *Gpihbp1^–/–^* mice have no overt pulmonary phenotypes (Beigneux et al., 2007; Olafsen et al., 2010). While *LPL* emerged relatively early in evolution and is present in teleosts (Holmes et al., 2011), GPIHBP1 evolved later and is confined to mammals (Holmes and Cox, 2012). This evolutionary delay raises the question of how lower vertebrates such as birds, fishes, and reptiles transport LPL to the capillary lumen (He et al., 2017b).

### Structure of GPIHBP1

#### Atypical Member of LU-Domain Protein Family

**FIGURE 3:**
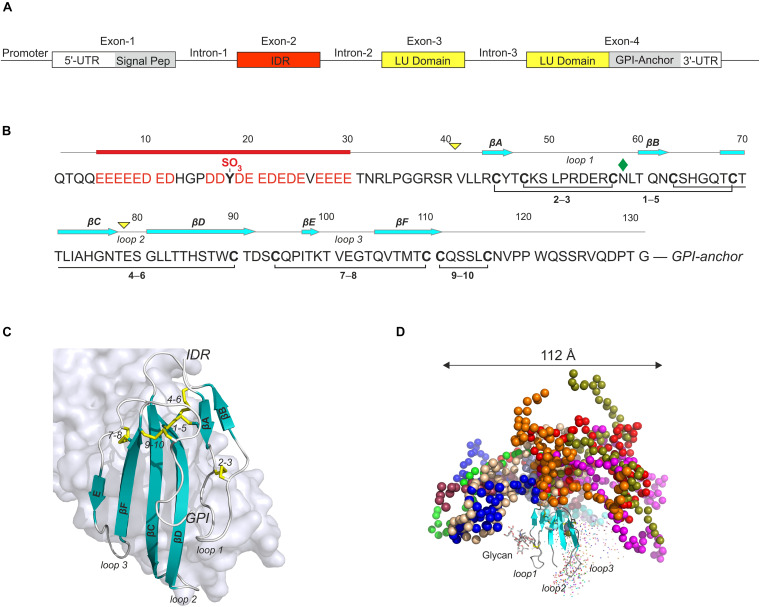
GPIHBP1 is an atypical member of the LU-domain gene family. **(A)** The organization of human *GPIHBP1* on chromosome 8q24. Unlike canonical members of the LU supergene family, which have three exons, *GPIHBP1* has an extra exon (IDR, red box) encoding an intrinsically disordered acidic N-terminal extension. **(B)** This extension contains 21 acidic residues and one sulfated tyrosine in the first 30 residues of mature GPIHBP1^1– 131^ (Kristensen et al., 2018) and it is denoted GPIHBP1’s acidic domain. Mature GPIHBP1^1– 131^ contains a disordered acidic domain followed by a canonical folded LU domain harboring five plesiotypic disulfide bonds (Leth et al., 2019a) and a C-terminal peptide terminating in a membrane anchoring glycosylphosphatidylinositol moiety. *Yellow triangles*, intron positions; *cyan arrows*, β-strand positions; *red bar*, acidic disordered region; *green diamond*, N-linked glycan. **(C)** The structure of GPIHBP1’s LU domain (cartoon) bound to LPL (light gray surface representation) [PDB 6E7K] (Birrane et al., 2019). The acidic domain is not shown, as it was not defined by the electron densities from the crystal diffraction and presumably forms a fuzzy complex with LPL’s basic patch. **(D)** The likely spatial distribution of the acidic domain in a model of GPIHBP1^1– 131^ generated by simulating data from small-angle X-ray scattering (Kristensen et al., 2018).

In humans, *GPIHBP1* is located on chromosome 8q24.3 in a small cluster of 11 genes that encode a Ly6/uPAR (LU) domain (Loughner et al., 2016; Leth et al., 2019a). *GPIHBP1* is an atypical member of the LU supergene family because it contains four exons (Figure 3A) rather than the usual three. The extra exon (exon-2) in *GPIHBP1* encodes a 40-residue, intrinsically disordered N-terminal extension with 21 acidic amino acids (Asp or Glu); this extension undergoes posttranslational O-sulfation of Tyr^18^ (Kristensen et al., 2018) and is denoted GPIHBP1’s acidic domain. Exons 3 and 4 in *GPIHBP1* encode the archetypical LU domain and a C-terminal signal peptide responsible for the covalent attachment of a GPI membrane anchor (Figure 3A).

Nascent human GPIHBP1 is synthesized by capillary endothelial cells as a 184-residue single-chain polypeptide. Posttranslational processing steps include (i) removal of N- and C-terminal signal peptides responsible for secretion and glycolipid anchoring, respectively, (ii) N-linked glycosylation of Asn^58^ (Kristensen et al., 2018), (iii) *O*-sulfation of Tyr^18^ (Kristensen et al., 2018), and (iv) formation of the five plesiotypical disulfide bonds defining the canonical LU domain (Cys^35^–Cys^69^, Cys^48^–Cys^57^, Cys^63^–Cys^90^, Cys^94^–Cys^110^, and Cys^111^–Cys^116^) (Figure 3B). The folded LU domain of mature GPIHBP1 (residues 42–109) adopts a three-fingered fold with a cysteine-rich core projecting three long β-hairpins (loops 1, 2, and 3) that assemble into a slightly curved central β-sheet (Figure 3C; Birrane et al., 2019; Leth et al., 2019a). It binds to LPL’s PLAT domain along the entire concave face of this central β-sheet, including all three loops, and burying 940 Å^2^ of its surface in the binding interface (Birrane et al., 2019). This binding pose aligns well with data from HDX-MS analyses (Mysling et al., 2016a) and site-directed mutagenesis (Beigneux et al., 2011; Voss et al., 2011)—and it explains why certain genetic variants of GPIHBP1 and LPL are associated with hypertriglyceridemia (Henderson et al., 1996, 1998; Surendran et al., 2012; Buonuomo et al., 2015; Pingitore et al., 2016) as they compromise the complementarity of the binding interface.

#### Intrinsically Disordered Acidic Domain in GPIHBP1

GPIHBP1 has a disordered, highly acidic N-terminal extension that has a decisive role in orchestrating the impact of LPL on intravascular lipid metabolism. This atypical extension was most likely acquired after duplication of an ancestral LU gene and the subsequent integration of a 40-residue polypeptide segment from *BCL11A* into *GPIHBP1* (Holmes and Cox, 2012). This polypeptide contains 21 negatively charged residues (Glu or Asp) and one tyrosine-*O*-sulfation (Kristensen et al., 2018) within a continuous stretch of 26 residues (Figure 3B). The disorder of the acidic domain in GPIHBP1 is evident from (i) disorder predictions, (ii) its aberrant elution profile from size exclusion chromatography, (iii*)* Kratky plots of small angle X-ray scattering (SAXS) analyses, (vi) its fast hydrogen-deuterium exchange profile, and (v) its absence from the crystal structure of the GPIHBP1•LPL complex (Mysling et al., 2016a; Kristensen et al., 2018; Birrane et al., 2019). Although GPIHBP1 is a relatively small protein (131 residues) with a mass of only 15.7 kDa, these features render it highly asymmetrical, with an N-terminal disordered acidic region, a stably folded disulfide-rich core LU domain, and a short C-terminal peptide that tethers the protein to the cell membrane via a glycolipid anchor (Figure 3). GPIHBP1’s disordered acidic domain most likely occupies a “mushroom-shaped space” 112 Å in diameter atop the LU domain (Kristensen et al., 2018; Figure 3D). This unique topology enables GPIHBP1 to rapidly bind LPL, stabilize its structure, and extract HSPG-bound LPL from a dynamic pool in the subendothelial space (Figure 1).

#### Kinetics of the LPL–GPIHBP1 Interaction

Kinetic analysis of the interaction between LPL and GPIHBP1 by surface plasmon resonance (SPR) studies is far from trivial because LPL is unstable and prone to aggregation. Nonetheless, available SPR studies agree that GPIHBP1 has two distinct interactions with LPL—a fast and transient electrostatic interaction between the acidic domain and LPL’s heparin binding sites and a slow and stable interaction between the folded LU domain and LPL’s PLAT domain (Reimund et al., 2015; Mysling et al., 2016a; Kristensen et al., 2018). In one study the SPR data was interpreted in favor of a complex binding model (Reimund et al., 2015), in which the two distinct binding sites on GPIHBP1 act independently allowing it to bridge two LPL molecules. In another study, the SPR data suggested that GPIHBP1 binds to a single LPL molecule and that the two binding sites cooperate to produce very fast association rates (*k*_*on*_ of 3 × 10^8^ M^–1^s^–1^) and moderate dissociation rates (*k*_*off*_ of 1 × 10^–2^ s^–1^) (Kristensen et al., 2018). That model was further validated by orthogonal methods such as native gels, size-exclusion chromatography coupled to small angle X-ray scattering, and X-ray crystallography (Kristensen et al., 2018, 2020a; Birrane et al., 2019). The ultrafast *k*_*on*_ for LPL binding is driven by electrostatic steering between GPIHBP1’s acidic domain and LPL’s large basic patch. Indeed, deletion of the acidic domain in GPIHBP1 or inclusion of a high salt concentration drastically decrease *k*_*on*_ but had little effect on *k*_*off*_ (Kristensen et al., 2018). This observation aligns well with the concept that an intrinsic protein disorder often underlies fast association rates by increasing the effective capture radius, by the lack of orientation restraints, and by exploiting long-ranged electrostatics (Ganguly et al., 2012).

The interplay between the fast interaction of LPL with the acidic domain of GPIHBP1 and the slower but more stable interaction with the LU domain enables GPIHBP1 to extract HSPG-bound LPL from the subendothelial space. However, the fast kinetics results in transient LPL–GPIHBP1 interactions, which probably is insufficient for a stable margination of large chylomicrons along the capillary endothelium, where they are subject to sheer force from blood flow. Yet, LPL•GPIHBP1 complexes are indispensable for the margination of TRLs at these sites. We speculate that simultaneous binding of several LPL•GPIHBP1 complexes are required to generate sufficient avidity to overcome the sheer stress. This possibility is consistent with clustering of GPIHBP1 in sphingomyelin-rich microdomains of the capillary endothelial cell plasma membrane, likely promoted by GPIHBP1’s membrane anchoring by GPI (Zurzolo and Simons, 2016). It was estimated that 40–50 LPL molecules must simultaneously engage each TRL to match the rate of triglyceride hydrolysis of TRLs in solution *in vitro* (Scow and Olivecrona, 1977).

#### Stabilizing Effect on LPL

Soon after the discovery of GPIHBP1 as an obligate binding partner for LPL (Beigneux et al., 2007) it became clear that binding of GPIHBP1 prevents spontaneous inactivation of LPL’s enzyme activity (Sonnenburg et al., 2009). This protective effect is primarily mediated by GPIHBP1’s acidic domain, which prevents the progressive unfolding of LPL’s α/β–hydrolase domain (Mysling et al., 2016a). Binding of the LU domain to LPL only marginally delays this spontaneous inactivation. The stabilizing effects of GPIHBP1 on LPL activity was confirmed by differential scanning fluorimetry, which showed that the low apparent melting temperature of LPL’s α/β–hydrolase domain (*T*_m_ of 34.8°C) is increased dramatically by GPIHBP1^1–131^ binding (*T*_m_ of 57.6°C) but only marginally by GPIHBP1^34–131^ binding (*T*_m_ of 37.7°C), underscoring the pivotal role of the acidic domain in this process (Leth-Espensen et al., 2021). Analysis of deuterium uptake in LPL bound to various GPIHBP1 variants showed that GPIHBP1’s acidic domain binds to a heparin binding motif (residues 279–293) at the interface between LPL’s hydrolase and PLAT domains (Mysling et al., 2016a). That particular interaction site is remarkable because it is close to (i) regions that are affected by disease-relevant missense mutations (e.g., p.Ser^259^Arg, p.Gly^409^Arg, and p.Glu^410^Val); (ii) the C-terminus of LPL, where truncation of two residues (p.Ser^447^X) is associated with beneficial effects in population studies; and (iii) the PCSK3 (furin) cleavage site in LPL (residues 296–299). The susceptibility of LPL to PCSK3 cleavage is impacted reciprocally by ANGPTL4 and GPIHBP1 binding (Lund Winther et al., 2021).

### Biology of GPIHBP1

With the discovery of GPIHBP1, the pathway for LPL transport and distribution in the intravascular unit changed radically (Young et al., 2019) and new etiologies for both inborn and acquired hypertriglyceridemia were outlined (Figure 1).

#### Subendothelial Partitioning of LPL

After secretion from parenchymal cells, *de novo* synthesized LPL remains attached to the cell surfaces through transient electrostatic interactions with HSPGs (Figure 1). In the absence of GPIHBP1, LPL accumulates in the sub-endothelial space, bound to HSPGs on parenchymal cells and the surrounding extracellular matrix (Figure 1, *inset*). In tissues from *Gpihbp1^–/–^* mice, LPL is neither uniformly distributed throughout the sub-endothelial spaces nor swept away by the lymph drainage. Under these conditions, LPL is surprisingly more abundant around the abluminal surface of capillaries (Davies et al., 2010; Kristensen et al., 2018). This migration of LPL from parenchymal cells to capillaries implies that it moves against the flow from capillary fluid extrusion, at least in the part proximal to the arteriolar connection. This partitioning of LPL in the subendothelial space may be driven by directed diffusion (Duchesne et al., 2012) along a charge gradient created by differences in the density or degree of sulfation of HSPGs in the sub-endothelial space. The final destination for this directed movement of LPL is likely collagen XVIII, one of the major HSPGs deposited in the vascular basement membrane (Gordts and Esko, 2018). Consistent with this hypothetical model, mice with collagen XVIII-deficiency (*Col18^–/–^*) have moderate hypertriglyceridemia and reduced plasma levels of LPL (Bishop et al., 2010). A moderate hypertriglyceridemia phenotype also occurs in humans who are homozygous for collagen XVIII deficiency (Knoblochs syndrome).

We propose that secreted LPL remains tethered to HSPGs by transient electrostatic interactions with LPL’s basic heparin-binding patch, which stabilizes LPL while allowing it to gradually migrate to the abluminal surface of capillaries (Figure 1B). At this location LPL forms a dynamic reservoir though transient interactions with collagen XVIII, from which it can be mobilized in-*cis* by binding to GPIHBP1—a process in which the asymmetrical topology of GPIHBP1 plays a central role (Kristensen et al., 2018). The disordered and polyanionic N-terminal serves as a “decoy” that transiently extracts LPL from collagen XVII. Subsequent interaction with GPIHBP1’s more stable LU-domain confers sufficient longevity to the complex allowing it to complete the extraction. We validated this cooperativity between GPIHBP1’s acidic and LU domains in a surrogate model based on SPR data (Kristensen et al., 2018). Heparin sulfate was immobilized on the sensor chip, creating a reservoir of loosely attached LPL, which was retained on the surface by fast association and dissociation events driven by electrostatics. Injections of GPIHBP1^1–131^ or GPIHBP1^34–131^ over that surface revealed that both variants bound to the dynamic pool of LPL molecules, but only intact GPIHBP1^1–131^ extracted HSPG-bound LPL (Kristensen et al., 2018). This finding was confirmed in a surrogate *in vivo* model (Allan et al., 2017b).

#### Margination of TRLs Along the Luminal Surface of Capillaries

Complexes between LPL and GPIHBP1 that are formed along the abluminal surfaces of the capillary endothelium are transcytosed in vesicles to the luminal surface (Davies et al., 2010; Figure 1B). This cross-endothelial transport is bidirectional and independent of caveolin-1, as shown *in vivo* with a fluorescently labeled monoclonal anti-GPIHBP1 antibody (Davies et al., 2012). This implies that GPIHBP1 can shuttle several LPL molecules to the luminal surface by repeated cycles of transcytosis. Within the capillary lumen, the LPL–GPIHBP1 complex is solely responsible for margination of circulating TRLs, primarily at special anatomical structures on the capillary surface that, by electron microscopy, appear as “thin meadows” between tufts of a thicker glycocalyx (Goulbourne et al., 2014). Once TRLs are marginated, the intravascular processing of their triglyceride content is extremely rapid (He et al., 2017a). The fast release of fatty acids by triglyceride hydrolysis of TRLs *in vivo* is consistent with the notion that engagement of multiple LPL–GPIHBP1 complexes is required to retain TRLs on the luminal endothelial surface. Of note, efficient binding of TRLs *in vitro* to membrane-tethered LPL–GPIHBP1 requires an accessible Trp-rich loop in LPL’s PLAT domain (Goulbourne et al., 2014). The necessity for avidity effects to tether TRLs along the luminal surface may explain the subsequent release of remnant particles, whose smaller size and greater curvature would limit the number of bound LPL–GPIHBP1 complexes per particle, resulting in insufficient binding strength to withstand the vascular shear force (Figure 1).

In the mammary gland, the polarized secretion of LPL to milk by the epithelium of lactiferous ducts does not require GPIHBP1. In this setting, LPL is produced primarily by the epithelial cells, but also by neighboring adipocytes (Jensen et al., 1991), and is transcytosed to the lumen of the mammary ducts by sortilin-related receptor (Klinger et al., 2016). Fat and casein micelles likely stabilize the secreted LPL in milk.

### Dysfunctional GPIHBP1 Causes Hypertriglyceridemia

After the discovery of GPIHBP1 as the obligate partner for LPL in intravascular lipolysis (Young et al., 2019), *GPIHBP1* is now included in the panel of five canonical driver genes that are routinely tested to identify patients with monogenic familiar chylomicronemia—along with *LPL*, *APOC2*, *APOA5* and *LMF1* (Brahm and Hegele, 2015; Baass et al., 2020).

#### Disease-Relevant Missense Variants in GPIHBP1

The proper folding of LU-domain proteins is generally sensitive to missense mutations of any of its plesiotypic cysteine residues or to deletion of its disulfide bonds (Figure 3B; Leth et al., 2019a, b). This sensitivity, combined with the high abundance of cysteine residues (10–15%), explains why the majority of disease-causing variants of GPIHBP1 affects plesiotypic cysteine residues (e.g., p.Cys^65^Tyr, p.Cys^65^Ser, p.Cys^68^Tyr, p.Cys^68^Gly, p.Cys^83^Arg, p.Cys^89^Phe) (Fong et al., 2016; Lima et al., 2021). In cell culture experiments, *GPIHBP1* variants with missense mutations affecting cysteine residues give rise to multimerized GPIHBP1 molecules on the cell surface that do not bind LPL (Beigneux et al., 2009, 2015). In one patient, a deleterious GPIHBP1 variant arose due a missense mutation introducing an extra cysteine residue (p.Ser^107^Cys) with a free unpaired thiol group (Plengpanich et al., 2014). Mice that are homozygous *Gpihbp1*^*C*63*Y*/*C*63*Y*^, equivalent to the p.Cys^65^Tyr *GPIHBP1* variant in humans, develop lifelong severe chylomicronemia accompanied by the mislocalization of LPL in the subendothelial spaces (Allan et al., 2017a). Despite their normal transcript levels these mice have very low levels of GPIHBP1 in their capillaries, which does not bind LPL, consistent with the compromised folding and expression of this variant.

Although the acidic domain constitutes 30% of the primary sequence of mature GPIHBP1, no gene variants linked to hypertriglyceridemia have been localized to this region. This uneven distribution probably reflects profound differences in the physiochemical properties of GPIHBP1’s two domains. First, the LU domain is relatively sensitive to missense mutations because of their adverse impact on folding (Beigneux et al., 2015), whereas the acidic domain is resilient due to its intrinsically disordered nature. Second, the LU domain exploits distinct hot-spot residues (e.g., Trp^89^) to interact with LPL (Beigneux et al., 2011; Birrane et al., 2019), whereas the acidic domain forms a fuzzy complex with the basic surface of LPL, probably driven by the average electrostatic field rather than by discrete and decisive binding events (Reimund et al., 2015). Thus, the majority of single missense variants in GPIHBP1’s acidic domain likely do not affect its impact on LPL function.

#### Autoantibodies Toward GPIHBP1

Recently, GPIHBP1 auto-antibodies were shown to cause late-onset chylomicronemia by blocking the interaction between LPL and GPIHBP1 in 22 patients with a hitherto unexplained form of acquired hypertriglyceridemia (Beigneux et al., 2017; Miyashita et al., 2020). The ability of GPIHBP1 to undergo bidirectional transcytosis with a monoclonal antibody as cargo (Davies et al., 2012) may exacerbate the syndrome by preventing renewed loading of GPIHBP1 with LPL in the subendothelial spaces (Figure 1D). Normal plasma triglyceride levels were restored in some of these patients by immunosuppressive treatments with mycophenolate mofetil or prednisolone, but rituximab appeared to induce a more frequent and persistent remission (Ashraf et al., 2020; Lutz et al., 2020; Miyashita et al., 2020).

## Angiopoietin-Like Protein Inhibitors of LPL Activity

**FIGURE 4:**
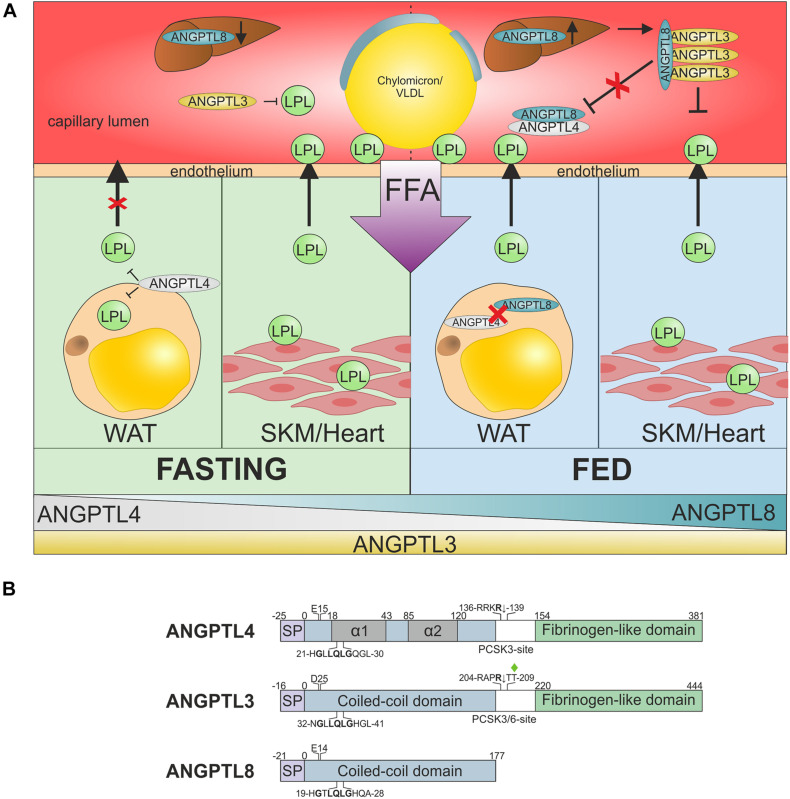
ANGPTL-3, −4, and −8 regulate nutrition-dependent TRL processing across tissues. **(A)** Suppression of LPL activity in oxidative and storage tissues by ANGPTL inhibition in the fed and fasted state. During fasting (*green*) or exercise, TRLs need to be directed away from storage in WAT toward oxidative tissues, such as heart and skeletal muscles (SKM). This is accomplished by (i) upregulation of ANGPTL4 in WAT, which suppresses LPL secretion and inactivates LPL in the sub-endothelial space, and (ii) downregulation of hepatic ANGPTL8 expression, which greatly reduces the potency of ANGPTL3-mediated LPL inhibition (Zhang, 2016; Chi et al., 2017; Cushing et al., 2017). Upon re-feeding (*blue*), the TRL flux must be rapidly reversed from oxidative tissues back to storage tissues. This transition is mediated by the rapid upregulation of ANGPTL8 expression in liver and WAT, combined with a decrease in ANGPTL4 expression in WAT (Oldoni et al., 2020). The resultant secretion of a hepatic ANGPTL3–ANGPTL8 complex mediates endocrine inhibition of LPL in oxidative tissues. The increased synthesis of ANGPTL8 in WAT may attenuate LPL inhibition by ANGPTL4 in an autocrine/paracrine manner that favors TRL processing in this tissue. For clarity GPIHBP1 is not shown. **(B)** Schematic representation of the domain architectures of ANGPTL-3, -4, and -8, showing the signal peptide (*SP*), coiled-coil domain (*blue*), and fibrinogen-like domain (*green*). The position of two α-helical segments in the coiled-coil domain of ANGPTL4 localized by HDX-MS are shown in *gray* (Mysling et al., 2016b; Leth-Espensen et al., 2021). The conserved segment in the coiled-coil region that is involved in LPL inhibition is highlighted (Lee et al., 2009). Two gene variants of ANGPTL4 and ANGPTL3 that are associated with reduced risk of ASCVD in epidemiological studies are highlighted (p.Glu^15^Lys and p.Asp^25^Asn).

In the past decade, genetic, epidemiologic, biochemical, and pharmacological studies have refined our understanding of nutrition-dependent inhibition of LPL in different tissues, which is responsible for partitioning TRLs between oxidative and storage organs (Figure 4A). The emerging theme is that ANGPTL4 regulates lipid uptake primarily in WATs by autocrine/paracrine inhibition of LPL in the fasted state, whereas an ANGPTL3–ANGPTL8 complex regulates lipid uptake in oxidative tissues by endocrine inhibition of LPL in the fed state (Dijk and Kersten, 2016; Zhang, 2016). As a result, lipid stores can be replenished in WAT during conditions of excess TRL supply (e.g., postprandially), and organs with high oxidative energy demand can receive sufficient lipids when TRLs are limited (e.g., during fasting, exercise, and cold exposure).

### ANGPTL4

ANGPTL4, also referred to as fasting-induced adipose factor, was originally cloned as a target gene under transcriptional control by the fatty acid–activated peroxisome proliferator–activated receptor (PPAR) α and PPARγ (Kersten et al., 2000; Yoon et al., 2000). ANGPTL4 is expressed at high levels in white and brown adipose tissue and liver and at lower levels in skeletal muscle, heart, intestine, and macrophages. The nutrition-dependent variation in expression of ANGPTL4 in adipocytes is regulated by the free fatty acid sensing transcription factor PPARγ.

#### Structure of ANGPTL4

Human ANGPTL4, a 50-kDa single-chain protein of 381 amino acids, has an N-terminal coiled-coil domain (residues 1–143) connected to a C-terminal fibrinogen-like domain by a linker region (Figure 4B). The three-dimensional structure of ANGPTL4 has not been determined, but the structure of its C-terminal fibrinogen-like domain was solved by X-ray crystallography (Biterova et al., 2018). The N-terminal coiled-coil domain is reported to contain a distinct mixture of monomers, dimers, and tetramers on the cell surface that are allegedly formed by covalent cross-linking of intermolecular disulfide bonds between Cys^51^ and Cys^55^ (Ge et al., 2004a, b; Yau et al., 2009; Yin et al., 2009; Makoveichuk et al., 2012). These studies found that the covalent oligomerization of ANGPTL4 is important for LPL inhibition; however, others showed that blocking disulfide formation by mutagenesis or chemical modification has little impact on LPL inhibition *in vitro* (Shan et al., 2009; Mysling et al., 2016b). A different oligomer state was reported for refolded coiled-coil domains expressed in *E. coli*, which adopts stable, elongated and flexible trimers (Gunn et al., 2021). This study did not address whether these trimers were assembled by intermolecular disulfides, as suggested previously (Sukonina et al., 2006). Nevertheless, the majority of ANGPTL4’s coiled-coil domain forms a 1:1 complex with ANGPTL8 in plasma (Chen et al., 2020). This observation may indicate that ANGPTL4 is either a monomer or that ANGPTL8 binding disrupts the oligomer state of ANGPTL4. More studies are required to settle this issue.

The linker region between the N- and C-terminal domains of ANGPTL4 contains a *bona fide* proprotein convertase cleavage motif (Figure 4B), and secreted ANGPTL4 is indeed cleaved at this site both in cultured cells and *in vivo* (Chomel et al., 2009; Yin et al., 2009; Lei et al., 2011). The liberated coiled-coil domain inhibits LPL more efficiently than intact ANGPTL4 (Yin et al., 2009; Chi et al., 2015). The inhibitory effect of ANGPTL4 critically depends on a small conserved motif including His^21^, Gln^25^ and Gln^28^ in the first α-helix of the coiled-coil domain (Figure 4B; Lee et al., 2009; Yau et al., 2009). The physiological importance of the first α-helix in ANGPTL4 is illustrated by the reduced risk for ASCVD conferred by the *ANGPTL4* variant p.Glu^15^Lys (Romeo et al., 2009; Dewey et al., 2016; Stitziel et al., 2016). This missense mutation lowers the helix propensity of that particular region by destabilizing the helix dipole momentum (Mysling et al., 2016b).

#### *Modus operandi* for ANGPTL4-Mediated LPL Inhibition

The molecular mechanism by which ANGPTL4 inhibits LPL activity is incompletely understood and controversial. One view holds that ANGPTL4 is a reversible non-competitive inhibitor of LPL (Lafferty et al., 2013; Gutgsell et al., 2019; Gunn et al., 2021), while an opposing view holds that ANGPTL4 *catalyzes* the irreversible unfolding of LPL’s α/β-hydrolase domain (Sukonina et al., 2006; Mysling et al., 2016b; Kristensen et al., 2020a, b; Leth-Espensen et al., 2021). This controversy probably stems from the inherent instability of LPL and from differences in the use of stabilizing excipients. A third view holds that ANGPTL4 inhibits LPL by promoting PCSK3-mediated cleavage of LPL at the cognate cleavage motif Arg–Ala–Lys–Arg^297^↓Ser–Ser separating the N- and C-terminal domains (Dijk et al., 2018); however, this regulation is probably linked to the mechanism of LPL unfolding (Lund Winther et al., 2021).

The reversible non-competitive inhibition model of LPL inactivation by ANGPTL4 is based on data from classical enzyme kinetic analyses. Initially, these analyses used 1-mM sodium deoxycholate as LPL stabilizing excipient and a soluble esterase substrate to monitor enzyme activity, which yielded a relatively high inhibition constant (*K*_*i*_) of 0.9–1.7 μM (Lafferty et al., 2013; Gutgsell et al., 2019). This is inconsistent with the observation that nanomolar concentrations of ANGPTL4 readily inhibit nanomolar levels of LPL (Sukonina et al., 2006; Sonnenburg et al., 2009; Mysling et al., 2016b; Kovrov et al., 2019; Chen et al., 2020; Nimonkar et al., 2020). Omitting sodium deoxycholate as excipient and using VLDL particles as substrate yielded a more reasonable inhibition constant (*K*_*i*_ of 3.2 nM), but the reaction kinetics still followed a reversible non-competitive inhibition model (Gunn et al., 2021). Several concerns about this model still pertain. First and foremost, the model does not explain why substoichiometric amounts of ANGPTL4 completely inhibit LPL in a time-dependent manner (Sukonina et al., 2006; Sonnenburg et al., 2009; Mysling et al., 2016b). Second, it does not explain why GPIHBP1 mitigates LPL inhibition by ANGPTL4 but does not restore the activity of LPL molecules already inhibited—as would be expected if the inhibition were reversible (Sonnenburg et al., 2009; Mysling et al., 2016b). Underscoring this concern, ANGPTL4 binds LPL–GPIHBP1 complexes at the same binding interface as free LPL, but the rate of inactivation is greatly diminished in the presence of GPIHBP1 (Chi et al., 2015; Leth-Espensen et al., 2021).

**FIGURE 5:**
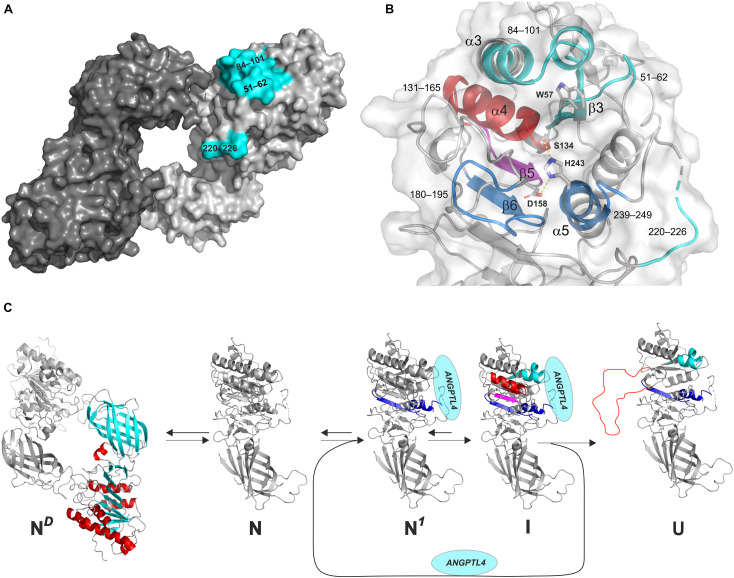
Model of the irreversible inhibition of LPL by ANGPTL4-catalyzed unfolding. **(A)** The ANGPTL4 binding site on LPL is highlighted in *cyan* on the surface representation of one of the protomers in the head-to-tail homodimer structure of LPL solved by X-ray crystal structure. **(B)** The architecture of the catalytic triad as a combined surface and cartoon representation. Regions involved in ANPTL4 binding (51–62 [β3], 84–101 [α3], and 220–226 [lid]) are *cyan*; regions where ANGPTL4 binding triggers increased dynamics by allostery are *blue* (180–195 [β6] and 239–249 [α5]) and *purple* (147–165 [β5]); and regions where irreversible unfolding represents the point-of-no-return inexorably leading to permanent LPL inhibition are *red* (133–145 [α4]). The catalytic triad is shown as sticks (Ser^134^, Asp^158^, and His^243^). **(C)** The reaction sequence of ANGPTL4-catalyzed unfolding of LPL’s α/β-hydrolase domain, leading to irreversible collapse of the catalytic pocket. N^*D*^: the native state of LPL homodimer; N: native state of LPL monomer; N^1^: native state of LPL in complex with ANGPTL4 with increased flexibility of α5 and β6 (*blue*); I: intermediate state of LPL with increased flexibility of β5 (*purple*) and reversible unfolding of α4 (*red*); U: unfolded state of LPL with irreversible unfolding of α4 (*red*).

The hallmark of the opposing model features an unprecedented *modus operadi* for enzyme inhibition: that ANGPTL4 inhibits LPL by catalyzing its irreversible unfolding. This model was pioneered by Sukonina et al. (2006), who insightfully dubbed ANGPTL4 a “molecular unfolding chaperone.” With the LPL homodimer model being prevalent at that time, they concluded that ANGPTL4 catalyzed the dissociation of stable LPL homodimers into unstable monomers that spontaneously lost activity. A decade later, HDX-MS studies largely confirmed these findings by showing that ANGPTL4 catalyzes the unfolding of LPL’s α/β-hydrolase domain (Mysling et al., 2016b). Subsequent HDX-MS studies showed that ANGPTL4 does in fact catalyze the unfolding of trapped LPL monomers as efficiently as LPL dimers—raising doubts as to the molecular mechanism of LPL inhibition by ANGPTL4 (Kristensen et al., 2020a). These studies indicated that ANGPTL4 most likely acts directly on LPL monomers rather than on LPL dimers. It is nonetheless formally possible that ANGPTL4 promotes the dissociation of LPL dimers if they are present. The binding site for ANGPTL4 is atop the entrance to the catalytic pocket, close to the LPL dimer interface (Gutgsell et al., 2019; Leth-Espensen et al., 2021). Thus, ANGPTL4-binding could dissociate LPL dimers into monomers by imposing steric constraints on the dimer assembly or by modifying the binding interface though conformational allostery (Figure 5A).

A more likely model is however that binding of ANGPTL4 to LPL monomers funnel these into an irreversible unfolding trajectory (Figure 5C; Leth-Espensen et al., 2021). The rapid equilibrium between monomers and dimers (Lookene et al., 2004) would drive the consumption of LPL dimers, if they were present. HDX-MS studies reveal that the binding of ANGPTL4 to LPL triggers a sequence of allosteric events that culminate in collapse of the catalytic pocket (Figure 5B). This irreversible unfolding of LPL is likely primed by conformational changes in one of the ANGPTL4 binding regions (β2–α3) combined with increased dynamics in two distant regions (α5 and β6), followed by the sequential unfolding of β5 and α4, with the latter event being the “point of no return” (Leth-Espensen et al., 2021). This model is consistent with time-dependent complete inhibition of LPL by substoichiometric amounts of ANGPTL4 (Sukonina et al., 2006; Sonnenburg et al., 2009; Mysling et al., 2016b). Since LPL unfolding catalyzed by ANGPTL4 resembles spontaneous unfolding, but is faster, ANGPL4 probably lowers the kinetic barrier for entering this unfolding trajectory (Mysling et al., 2016a, b; Kristensen et al., 2020a, b; Leth-Espensen et al., 2021). Indeed, ANGPL4 lowers the apparent *T*_m_ of LPL unfolding by about 20°C, as shown by differential scanning fluorimetry (Leth-Espensen et al., 2021). The catalyzed unfolding of LPL also provides a molecular mechanism for the protective effect of GPIHBP1 and for the accelerated PCSK3 cleavage of LPL in the presence of ANGPTL4 (Lund Winther et al., 2021).

#### GPIHBP1 Counteracts ANGPTL4 Inhibition

Lipoprotein lipase inhibition by ANGPTL4 is abrogated at 20°C when LPL is bound to GPIHBP1, but this effect is less pronounced at 37°C (Sonnenburg et al., 2009; Chi et al., 2015; Mysling et al., 2016b; Nimonkar et al., 2020; Shetty et al., 2020). The temperature-dependent protection of LPL by GPIHBP1 is consistent with the notion that ANGPTL4-catalyzed LPL inactivation is driven by lowering the kinetic barrier to unfolding of its α/β-hydrolase domain (Leth-Espensen et al., 2021). As mentioned previously, the stability of LPL increases dramatically in the presence of GPIHBP1 (raising *T*_m_ from 34.8°C to 57.6°C), while the presence of ANGPTL4 lowers *T*_m_ by 20°C to less than 15°C for LPL alone and to 36.6°C for LPL–GPIHBP1 complexes (Leth-Espensen et al., 2021). At normal body temperature, free LPL is borderline stable (*t*_1/2_ ∼180 s) and is metastable in the presence of ANGPTL4 (*t*_1/2_ < 5 s), while LPL in LPL–GPIHBP1 complexes is stable and is borderline stable in the presence of ANGPTL4 (*t*_1/2_ ∼70 s). This dependency would favor preservation of GPIHBP1-bound LPL activity along the capillary lumen.

The asymmetrical structure of GPIHBP1 is essential for this protective effect. In the presence of ANGPTL4, GPIHBP1^1–33^ and GPIHBP1^34–131^ do not effectively preserve LPL activity, either alone or in combination (Mysling et al., 2016b). Likewise, only the binding of intact GPIHBP1 confers significant thermostability on LPL’s α/β-hydrolase domain (Leth-Espensen et al., 2021). These findings underscore the importance of the two-point tether mechanism in stabilizing the structure of LPL and regulating its activity.

#### ANGPTL4 Facilitates LPL Cleavage by PCSK3

Studies of adipocytes from wild-type and *Angptl4^–/–^* mice show that ANGPTL4 reduces LPL secretion by promoting its intracellular degradation by PCSK3 cleavage in late endosomes or in the *trans*-Golgi compartment (Dijk et al., 2016, 2018). However, it was unclear how ANGPTL4 sensitizes LPL to PCSK3 cleavage. Studies with purified components showed that substoichiometric amounts of ANGPTL4 accelerate LPL cleavage, while GPIHBP1 slows the cleavage (Lund Winther et al., 2021). Thus, the allosteric effect of ANGPTL4 on LPL conformation makes the cleavage site for PCSK3 more accessible, whereas binding of GPIHBP1 makes it less accessible, either by physical shielding through a transient interaction with the acidic domain or more likely by stabilizing a native LPL conformation in which this site remains cryptic.

#### Genetic Variants in ANGPTL4 and Pharmacological Intervention

Genetic and epidemiologic studies of genes associated with improved intravascular triglyceride hydrolysis identified a common *ANGPTL4* variant with a minor allele frequency of 2–3% in Caucasians. Homozygosity for this variant, denoted p.Glu^15^Lys or E40K, is associated with hypotriglyceridemia and reduced risk for ASCVD (Romeo et al., 2007, 2009; Dewey et al., 2016; Helgadottir et al., 2016; Stitziel et al., 2016). This variant lowers the helix-forming propensity of residues 15–45 (Mysling et al., 2016b), the region involved in LPL binding (Figure 4B), and impairs LPL inhibition (Shan et al., 2009; Yin et al., 2009; Mysling et al., 2016b). Although this genetic variant did not have aberrant PCSK3 cleavage, oligomerization or secretion, no N-terminal fragments accumulated in the medium of transfected cell cultures and in sera from transgenic mice (Yin et al., 2009) —probably because this variant destabilizes the N-terminal fragment, rendering it more prone to proteolytic degradation.

Since the p.Glu^15^Lys *ANGPTL4* allele lowers susceptibility to ASCVD by reducing repression of LPL activity, ANGPTL4 might seem to be a promising target for therapeutic management of dyslipidemia. Indeed, monoclonal anti-ANGPTL4 antibodies that abolish LPL inhibition lowered plasma TRL levels in mice. However, there were adverse side effects, including severe mesenteric lymphadenitis in mice fed a high-fat diet (Desai et al., 2007; Dewey et al., 2016). This was clearly a direct consequence of targeting ANGPTL4, as *Angptl4^–/–^* mice fed a diet high in saturated fat have a lethal phenotype characterized by severe inflammation and accumulation of foam cells in the mesenteric lymph nodes (Lichtenstein et al., 2010). Not surprisingly, interest in developing a systemic pharmacological targeting of ANGPTL4 waned, and the focus shifted to the alternative pathway of LPL inhibition, centering on the endocrine effect of ANGPTL3-ANGPTL8 in oxidative tissues.

### ANGPTL3 and ANGPTL8

In 2002, ANGPTL3 was linked to lipid metabolism when an *Angptl3* variant with a premature stop codon was identified as the sole cause of hypolipidemia in inbred KK/San mice (Koishi et al., 2002). ANGPTL3 is predominantly synthesized by hepatocytes. *Angptl3* expression is regulated by the oxysterol-activated liver X receptor and is largely independent of the nutrition status (Ge et al., 2005). Please consult the review by Dr. Kersten for a comprehensive review on ANGPTL3 (Kersten, 2017).

A decade later ANGPTL8 was shown to be a new endocrine inhibitor of LPL activity, but only in complex with ANGPTL3 (Quagliarini et al., 2012; Ren et al., 2012; Zhang, 2012). ANGPTL8 is primarily expressed in the liver, adipose tissues, and adrenal gland. Unlike ANGPTL3, ANGPTL8 expression is tightly controlled by nutrition status and is highly upregulated by feeding. The pivotal role of ANGPTL8 in plasma lipid homeostasis is illustrated by the decline in circulating triglyceride levels after refeeding of fasted *Angptl8^–/–^* mice compared to wild-type mice—a difference that was not observed during fasting (Wang et al., 2013). Thus, LPL activity is primarily controlled by ANGPTL4 in WATs and by ANGPTL3/ANGPTL8 in oxidative tissues, enabling delivery of TRLs across tissues to meet the energy demands of various nutrition states (Figure 4A; Zhang, 2016).

#### Oligomer State of ANGPTL3 and Cleavage by PCSK3 and PCSK6

Human ANGPTL3, a 62-kDa single-chain glycoprotein of 444 amino acids, has the same domain composition as ANGPTL4*—*an N-terminal coiled-coil domain (residues 1–143) and a C-terminal fibrinogen-like domain connected by a linker region (Figure 4B). When the coiled-coil domain of ANGPTL3 is expressed in *E. coli*, the refolded protein forms a mixture of elongated trimers and hexamers that do not interconvert (Gunn et al., 2021). Eukaryotic expression of full-length ANGPTL3 also yields a heterogeneous high-molecular-weight complex with an average mass of 440 kDa (Ge et al., 2005). Notably, ANGPTL3 forms a 3:1 complex with ANGPTL8 when they are co-expressed in cell culture or when they are isolated from human serum, and this complex is biologically relevant (Chen et al., 2020). The propensity of ANGPTL3 to form defined oligomers either alone or in complex with ANGPTL8 may have a bearing on their shared evolutionary origin—ANGPTL8 is a paralog of ANGPTL3 that arose before to the split into the mammalian branch by a gene duplication of an ancestral DOCK gene hosting an *ANGPTL* gene in one of its introns (Quagliarini et al., 2012).

ANGPTL3 can be cleaved *in vivo* by PCSK3 (intracellular) or by PCSK6 (on the cell surface) at its proprotein convertase cleavage motif located in the linker region (Figure 4B; Ono et al., 2003; Jin et al., 2007; Essalmani et al., 2013). Cleavage by PCSK3 is prevented by O-linked glycosylation of Thr^209^ (Figure 4B), which is added by the liver specific polypeptide GalNAc-transferase-2, and the occurrence of this modification controls the level of PCSK3 cleavage (Schjoldager et al., 2010). The biological significance of this cleavage is uncertain, although the released N-terminal coiled-coil domain of ANGPTL3 is a more efficient inhibitor of endothelial lipase (Jin et al., 2007), but not of LPL activity (Ono et al., 2003; Chi et al., 2017).

#### ANGPTL3 Forms an LPL Inhibitory Complex With ANGPTL8

Several studies with purified proteins or cell culture models show that ANGPTL3 inhibits LPL activity less efficiently than ANGPTL4, but the mechanism is unclear (Shan et al., 2009; Sonnenburg et al., 2009; Chi et al., 2017; Kovrov et al., 2019; Chen et al., 2020). Nevertheless, a single study found that ANGPTL3 was a potent inhibitor of LPL with an inhibition constant *K*_*i*_ of only 7.5 nM, twice that measured for ANGPTL4 (Gunn et al., 2021). The reason for this discrepancy in potency is unclear (Gunn et al., 2021). The need for supra-physiological concentrations of ANGPTL3 to inhibit LPL *in vitro* was enigmatic until it was realized that ANGPTL3 forms a complex with ANGPTL8 during synthesis in hepatocytes and that this complex is 20–100-fold more active than ANGPTL3 (Chi et al., 2017; Kovrov et al., 2019; Chen et al., 2020). Given (i) that the ANGPTL3–ANGPTL8 complex is the relevant inhibitor of LPL; (ii) that ANGPTL3 and ANGPTL8 both are synthesized in the liver; and (iii) that ANGPTL8 expression is induced by feeding outlined a mechanism for differential regulation of TRL processing in different tissues dependent on nutritional cues (Figure 4A). An additional layer of complexity to the regulation of LPL activity was recently added by the finding that ApoA5 bound tightly to the ANGPTL3–ANGPTL8 complex *in vitro* and *in vivo* and this binding impaired the capacity of the complex to inhibit LPL (Chen et al., 2021).

The molecular mechanisms for LPL inhibition by ANGPTL3 and ANGPTL3–ANGPTL8 complexes remains largely unknown. One study finds that ANGPTL3 efficiently inhibits LPL *via* reversible non-competitive inhibition (Gunn et al., 2021). Another study finds that ANGPTL3 catalyzes the unfolding of LPL’s catalytic domain, albeit less efficient than ANGPTL4 (Mysling et al., 2016b). A third mechanism for LPL inhibition by ANGPTL3 is that it sensitizes LPL to PCSK3 or PCSK6 cleavage on the cell surface in cell culture experiments (Liu et al., 2010). Of note, this PCSK3 cleavage of LPL on the cell surface is promoted by ANGPTL3, allegedly even in the presence of GPIHBP1. Both inhibition of LPL activity and sensitization to PCSK3 cleavage was enhanced by the ANGPTL3–ANGPTL8 complex (Jin et al., 2021). As judged by HDX-MS, the binding site on LPL for the ANGPTL3–ANGPTL8 complex coincide partly with that delineated for ANGPTL4 (Gutgsell et al., 2019; Jin et al., 2021; Leth-Espensen et al., 2021). In future studies, it would be interesting to assess by HDX-MS, whether the ANGPTL3–ANGPTL8 complex induces the same allosteric unfolding of LPL as ANGPTL4 and to define the precise roles for ANGPTL3 and ANGPTL8 in binding and inactivation of LPL. One study proposes that the inhibitory motif in the first α-helix of ANGPTL8 (Figure 4B) is responsible for LPL inhibition by the ANGPTL3–ANGPTL8 complex (Haller et al., 2017). In that model, the inhibitory motif is cryptic in ANGPTL8 but is exposed and active when ANGPTL8 is in complex with ANGPTL3. Additional high-resolution structural data are required to further substantiate this interesting model.

#### Genetic Variants and Pharmacological Intervention

Genetic studies found that loss-of-function variants in *ANGPTL3* are associated with reduced plasma levels of triglycerides, low-density lipoprotein cholesterol, and high-density lipoprotein cholesterol resulting in a reduced risk of ASCVD (Willer et al., 2008; Romeo et al., 2009; Musunuru et al., 2010; Lotta et al., 2018). One of these rare loss-of-function variants in *ANGPTL3* (p.Asp^25^Asn or D41N) resembles an *ANGPTL4* variant (p.Asp^15^Lys or E40K) inasmuch as both SNPs eliminate the negative side-chain in the start of the α-helix harboring the LPL binding motif (Figure 4B). The beneficial effects on plasma lipoprotein profiles by reduced ANGPTL3 activity were largely replicated in mice by pharmacological interventions using inhibitory monoclonal antibodies or antisense oligonucleotides (Gusarova et al., 2015; Dewey et al., 2017; Graham et al., 2017).

Prompted by these promising observations, pharmaceutical companies are currently developing new interventions strategies designed to lower the activity of ANGPTL3. Currently, two different strategies are being pursued; one based on a human monoclonal anti-ANGPTL3 antibody (evinacumab) that interferes with ANGPTL3s ability to inhibit LPL activity (Dewey et al., 2017; Raal et al., 2020; Rosenson et al., 2020) and another based on gene silencing using antisense nucleotides (AKCEA-ANGPTL3-LRx) that targets *ANGPTL3* mRNA (Graham et al., 2017). So far, safety and efficacy profiles of both drugs show promise in clinical trials for management of severe hypercholesterolemia (Mohamed et al., 2021).

## Perspectives

The last decade has brought an increased clarity into the molecular mechanisms driving intravascular lipolysis. This new insight combines three-dimensional protein structures and their conformational dynamics with the compartmental regulation of LPL activity in the vascular unit across tissues. Despite this advancement, the next decade will likely bring additional new insights into the mechanism by which the ANGPTL3-ANGPTL8 complex regulates LPL activity and how ApoA5 modulates this regulation.

## Author Contributions

MP wrote the manuscript with inputs from all authors. KK prepared the figures. All the authors contributed to the article and approved the final version.

## Conflict of Interest

The authors declare that the research was conducted in the absence of any commercial or financial relationships that could be construed as a potential conflict of interest.

## Footnotes

1 Throughout this review, amino acid numbering starts with the first residue of the mature protein.

## Abbreviations

ANGPTL: angiopoietin-like
ASCVD: atherosclerotic cardiovascular disease
GPIHBP1: glycosylphosphatidylinositol-anchored high density lipoprotein–binding protein 1
HDX-MS: hydrogen–deuterium exchange mass spectrometry
HSPG: heparan sulfate proteoglycan
IDR: intrinsically disordered region
LMF1: lipase maturation factor 1
LPL: lipoprotein lipase
PLAT: polycystin-1, lipoxygenase, and alpha toxin
PPAR: peroxisome proliferator–activated receptor
SAXS: small angle X-ray scattering
Sel1L: Sel-1 suppressor of Lin-12-like 1
SDC-1: syndecan-1
TRL: triglyceride-rich lipoprotein
VLDL: very low-density lipoprotein.
